# Unpleasant odors compared to pleasant ones cause higher cortical activations detectable by fNIRS and observable mostly in females

**DOI:** 10.1063/5.0231217

**Published:** 2025-01-07

**Authors:** Anna Maria Monciatti, Maddalena Lapini, Jessica Gemignani, Gabriele Frediani, Federico Carpi

**Affiliations:** 1 Biomedical Engineering Unit, Department of Industrial Engineering, University of Florence, 50121 Florence, Italy; 2 Department of Developmental and Social Psychology, University of Padua, 35131 Padova, Italy; 3 Padova Neuroscience Center, Padova, Italy; 4 IRCCS Fondazione don Carlo Gnocchi ONLUS, 50143 Florence, Italy

## Abstract

Olfactory perception can be studied in deep brain regions at high spatial resolutions with functional magnetic resonance imaging (fMRI), but this is complex and expensive. Electroencephalography (EEG) and functional near-infrared spectroscopy (fNIRS) are limited to cortical responses and lower spatial resolutions but are easier and cheaper to use. Unlike EEG, available fNIRS studies on olfaction are few, limited in scope, and contradictory. Here, we investigated fNIRS efficacy in assessing the hedonic valence of pleasant and unpleasant odors, using ten channels on each hemisphere, covering the orbitofrontal cortex and adjacent areas involved in olfactory and cognitive tasks. Measurements on 22 subjects (11 males and 11 females) showed statistically significant higher increases in oxygenated hemoglobin concentration for the unpleasant odor, compared to the pleasant one (mean difference = 1.025 × 10^−1^ *μ*M). No difference in activation was found between the hemispheres. Conversely, differences were observed between the sexes: for the first time, we show that higher activations for the unpleasant odor relative to the pleasant one are detectable by fNIRS in females (mean difference = 1.704 × 10^−1^ *μ*M), but not in an equal-sized and equal-age group of males. Moreover, females had greater activations relative to males for the unpleasant odor (mean difference = 1.285 × 10^−1^ *μ*M). Therefore, fNIRS can capture peculiarities of olfactory activations, highlighting differences between odors with opposite valence and between sexes. This evidence positions fNIRS next to EEG as suitable technologies for cortical investigations of olfactory perception, providing complementary information (late and early response components, respectively), with lower costs and easier operation (albeit at lower resolutions) compared to fMRI.

## I. INTRODUCTION

A significant characteristic of olfaction, differentiating it from other senses, is a robust association to hedonic valences.^1^ The hedonic assessment of odors is so important during human olfactory perception that pleasantness represents a fundamental dimension while characterizing olfactory stimuli.^2^ For instance, in experiments on the classification of odors, subjects spontaneously used pleasantness as the primary criterion of discrimination.^3^ In general, we classify odors as pleasant or unpleasant when their hedonic valence is positive or negative, respectively.^4^

Through their hedonic significance, odors can influence mood, stress, and anxiety;^5^ indeed, unlike visual or auditory stimuli, odors (as well as tastes) are intimately linked to intense affective reactions.^1^

Such a close relationship between olfaction and emotions is a logical consequence of the fact that both processes share their origin in several limbic regions.^6^ Accordingly, to study olfactory perception, it is necessary to investigate activations of those deep cerebral regions. This is typically accomplished using functional magnetic resonance imaging (fMRI), which utilizes the blood oxygen level dependent (BOLD) effect to map brain activity, at high spatial resolutions.^7^ However, accessibility to this technology is limited by its operational complexity and high costs, which can be excessive for many research institutions and small healthcare organizations.

To overcome those drawbacks, more easily accessible and cost-effective technologies, such as electroencephalography (EEG) and functional near-infrared spectroscopy (fNIRS), are of interest. Indeed, they are employed to that aim, although at the cost of limiting the investigation to a much lower spatial resolution, as well as to cortical regions only, as EEG and fNIRS cannot scan deeper portions of the brain. This prevents detection of early olfactory responses, which primarily originate from subcortical regions. Indeed, the areas initially involved in olfactory processes include the piriform cortex, amygdala, insula, and hippocampus, which are too deep to be detected by EEG and fNIRS. Nonetheless, cortical regions, such as the orbitofrontal cortex (OFC), middle and inferior frontal gyri, and inferior parietal lobule, are known to be implicated in later stages of olfactory processes.^5^ Therefore, they are a target of studies on olfaction accomplished with EEG and fNIRS.

In particular, EEG, which has a higher temporal resolution compared to fMRI, has been used in numerous studies on olfactory event-related potentials (ERP).^8,9^ Early negative components have been linked to olfactory sensory thresholds and odor identification, while late positive components have been associated with cognitive processing, in terms of evaluation and classification of odors based on novelty, familiarity, pleasantness, or significance.^10–13^

Despite the significant potential of EEG measurements, assessing olfactory ERP accurately is not trivial. Indeed, as they typically show up within a few hundred milliseconds, their evaluation can be significantly affected by the uncertainty on the instant at which olfactory receptors begin to be stimulated. This is due to the fact that olfactory stimuli have to necessarily be delivered by releasing odorant molecules from external reservoirs, such that there is a delay between the instant of release and the instant of binding to odorant receptors. Such a delay can vary, depending on the experimental conditions (such as release method, presence/size of conduits, and odorant diffusivity) and the type of sniffing action exerted by the subject (passive or active).

On the other hand, different features characterize fNIRS, which is used to monitor localized changes in oxygenated (HbO) and deoxygenated (HbR) hemoglobin.^14^ Like fMRI, it analyzes a hemodynamic response, whose duration is two orders of magnitude longer than that of olfactory ERP (orders of 10 and 0.1 s, respectively). In that respect, fNIRS appears as an interesting alternative to fMRI for investigating olfactory late processing by cortical regions (albeit at lower resolutions).

Accordingly, the motivation of this work was to elucidate how fNIRS performs in such a task, particularly in relation to the cortical processing of the hedonic valence of odors.

Indeed, this is not clear from the literature, as, to date, fNIRS studies on olfactory perception have been relatively few, rather limited in scope, very heterogeneous in the channel number and montages, and often contradictory.^15^ For instance, Ishimaru *et al.*^16^ used two channels on the OFC to record fNIRS responses to rose-, sweat-, and peach-like odors in ten adults, reporting greater activation in the right hemisphere. Harada *et al.*^17^ analyzed responses to strawberry essence and vanilla essence in 13 subjects over the frontal, temporal, parietal, and occipital regions, finding the highest activations in frontal regions, with no significant differences between the hemispheres. Kokan *et al.*^18^ used four channels on the OFC to measure responses to rose- and lemon-like odors in 14 females, reporting significant activations (total hemoglobin) in the left hemisphere; however, when the task consisted in the identification of the odors, the subjects who successfully accomplished it were reported to have more pronounced activations in the right hemisphere. In a later study, Igarashi *et al.*^19^ assessed, with two channels, responses to rose and orange essential oils from the prefrontal cortex in 20 females, reporting decreased activations in the right hemisphere, but no significant changes in the left one. Moein *et al.*^20^ employed four channels on the forehead to record responses to garlic and strawberry in 17 subjects, although without any investigation about possible lateralization effects. Therefore, the fNIRS literature presents contrasting findings on how the two hemispheres respond in general to olfactory stimuli.

Moreover, other incongruences emerging from the fMRI literature concern how the two hemispheres process the hedonic perception of odors. For instance, Royet *et al.*^6^ reported that, during exposure to a pleasant odor, the left hemisphere showed a more pronounced involvement than the right one. In contrast, in other studies, the right medial hemisphere was reported to encode pleasantness, whereas the left lateral hemisphere was reported to encode unpleasantness.^21,22^ Other investigations also reported higher activation in the right (orbitofrontal) cortex for a pleasant smell, but also described bilateral activations for unpleasant odors.^23–25^ Differently, Bensafi *et al.*^26^ reported that odors activated both hemispheres without lateralization, although unpleasant odors induced more activity than pleasant ones in the posterior OFC. Likewise, Rolls *et al.*^4^ indicated a lack of lateralization, although they reported that pleasant odors activated a medial region of the rostral OFC, whereas unpleasant odors induced more significant activation of a lateral region of the left rostral OFC.

Another aspect where the literature presents contrasting information is how the hedonic evaluation of odors is influenced by the subjects' sex. Several questionnaire-based investigations have indicated that in females the sensitivity to odors is higher.^27–30^ Furthermore, Olofsson and Nordin^31^ reported larger EEG amplitudes for females. However, other studies have reported a lack of differences between the sexes. For instance, although Lundström and Hummel^32^ reported larger EEG amplitudes and longer latencies on the left hemisphere for females and on the right one for males, they did not find sex-related differences in olfactory sensitivity or hedonic ratings of odors. Similarly, other questionnaire-based investigations did not obtain evidence of sex-related differences.^33–35^

In summary, evidence reported to date (gathered with a diversity of methods) is in general not consistent regarding possible lateralization effects, and possible differences between sexes, during the hedonic perception of odors.

In consideration of this, as well as of the limited number of investigations that have explored so far the potentialities of fNIRS in this field, here we present a systematic study, having the following three purposes: first, confirming that two odors with opposite hedonic valence (pleasant and unpleasant) can elicit significantly different activations detectable by fNIRS; second, clarifying whether the two odors can elicit significantly different activations in the two hemispheres, detectable by fNIRS; third, clarifying whether the two odors can elicit significantly different activations in males and females, detectable by fNIRS.

Our study was focused not only on the OFC, which fMRI investigations have shown to be particularly involved in odor perception and identification,^6,36–39^ but also on other adjacent cortical areas, to broaden the region of investigation, as detailed in Sec. II.

## II. EXPERIMENTAL SETUP AND OLFACTORY STIMULATION PROTOCOL

The study was conducted using three odorants, differing in their hedonic valence, classified as unpleasant, pleasant, and neutral. The unpleasant odorant was a sulfurous salt–bromine–iodine thermal water (Acqua di Sirmione, Menarini, Italy) with a characteristic sulfur smell, resembling rotten eggs. The pleasant odorant consisted of an orange essential oil (Citrus arantium L., Oligea Srl, Italy). The neutral odorant was mineral water.

The subjective hedonic perception of the odors generated by those odorants was assessed among the participants. Each subject was asked to classify each odor during the tests, choosing among unpleasant, pleasant, and neutral, as detailed later.

**FIG. 1.**
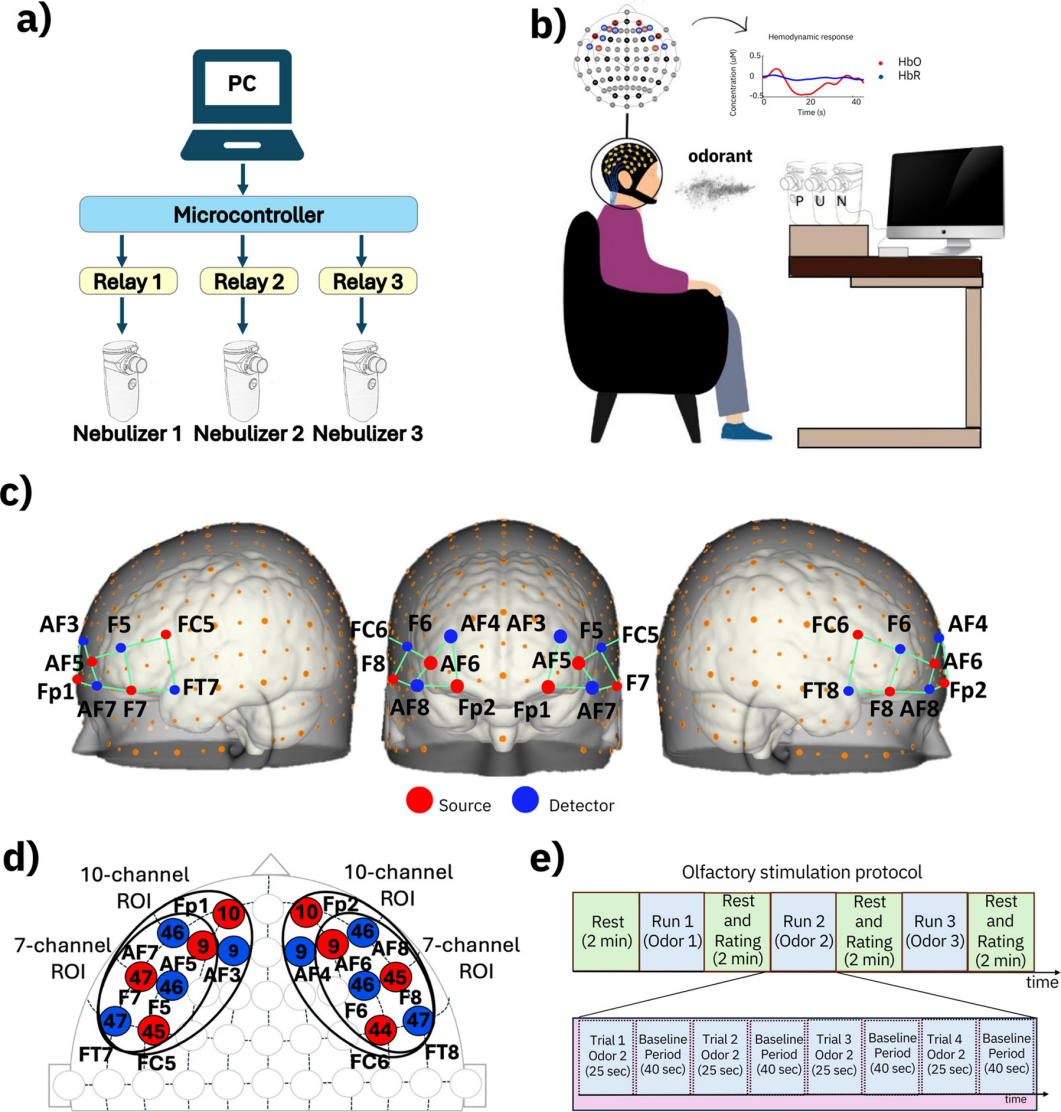
Experimental setup and olfactory stimulation protocol: (a) diagram of the olfactory stimulation system, consisting of three nebulizers, three relays, and a microcontroller. (b) Diagram of the setup. (c) 3D anatomical maps of the optodes montage, where sources and detectors are represented as red and blue dots, respectively. (d) 2D anatomical map of the optodes montage according to the 10–5 system; the numbers inside each optode indicate the cortical Brodmann area located underneath. (e) Stimulation protocol, showing the timing of the odor release phases and rest phases.

The olfactory stimulation was delivered using three mesh nebulizers, as schematically shown in Figs. 1(a) and 1(b). The nebulizers were modified so that they could independently be switched on and off via an external signal, as detailed in Methods.

Among 32 adult subjects initially tested, 22 of them (11 females and 11 males) were retained for this study, as detailed in Methods. Their fNIRS signals, i.e., the variations of their cortical concentrations of HbO and HbR in response to olfactory stimulations with the odorants described above, were recorded and processed as detailed in Methods.

In particular, the measurements were performed using, for each hemisphere, eight optodes (four emitters and four detectors), which were mounted according to the layout depicted in Fig. 1(c) (see Sec. VI for details). The optodes defined ten fNIRS channels on the left hemisphere (F7–F5, F7–AF7, F7–FT7, AF5–F5, AF5–AF7, AF5–AF3, Fp1–AF7, Fp1–AF3, FC5–F5, and FC5–FT7) and ten symmetrical fNIRS channels on the right hemisphere (F8–F6, F8–AF8, F8–FT8, AF6–F6, AF6–AF8, AF6–AF4, Fp2–AF8, Fp2–AF4, and FC6–FT8). The optodes approximately covered cortical areas (both in the OFC and adjacent areas) that are known to be explicitly relevant to olfactory processing, as well as adjoining areas, which were included because of their relevance to cognitive processing.^40^ In particular, as indicated in Fig. 1(d), the optodes were above the following Brodmann areas (BA): BA47 (part of the orbital cortex, mostly deputed to the hedonic valence of olfaction and to memory and emotion), BA45 (part of Broca's area, mostly deputed to speech, chosen for its closeness to the areas involved in olfaction and cognitive tasks), BA44 (part of Broca's area, mostly deputed to speech and motor action of fingers and toes, chosen for its proximity to the regions involved in olfaction), BA46 (part of the middle frontal cortex, mostly involved in motivation, attention, and cognitive tasks), BA09 (part of the dorsolateral prefrontal cortex, which plays a role in working memory and higher cognitive processes), and BA10 (part of the frontopolar area, which is involved in central executive functions).^40^

The analysis of the fNIRS signals gathered from the ten channels was conducted according to two distinct regions of interest (ROIs): a ten-channel ROI, consisting of all ten channels on each hemisphere, and a seven-channel ROI, which grouped a subset of seven channels (covering the OFC and adjacent areas) on the right hemisphere (F8–F6, F8–AF8, F8–FT8, AF6–F8, AF6–AF8, FC6–F6, and FC6–FT8) and seven symmetrical channels on the left hemisphere (F7–F5, F7–AF7, F7–FT7, AF5–F5, AF5–AF7, F5–FC5, and FT7–FC5). The optodes of the seven-channel ROI were selected because they covered the BA explicitly involved in olfactory processing (BA47) and four nearby areas (BA46, BA45, BA44, and BA09), which are indicated in Fig. 1(d).

Each subject received olfactory stimulations with the three odorants, which were presented one at a time, in random order. For each odorant, the subject experienced a sequence of four trials of olfactory stimulation, where each trial lasted 25 s, and was separated by the subsequent trial by an inter-stimulus rest phase (no odor provided), lasting 40 s, according to the stimulation protocol represented in Fig. 1(e).

The adopted durations of each trial (25 s) and the rest phase (40 s) were consistent with typical values reported in the literature.^15^ In particular, the duration of each trial was defined as a trade-off between two opposite needs: it had to be sufficiently long, to comply with the characteristic duration of OFC hemodynamic responses (order of magnitude of 10 s), but it also had to be sufficiently short, to avoid olfactory adaptation.

After the completion of the four trials for any given odorant, the subject was given a 2-min break, during which they completed a questionnaire. The questionnaire aimed to assess the hedonic valence of the odor just tested, by rating it according to a five-point Likert scale, ranging from −2 (very unpleasant) to +2 (very pleasant). An odor was classified as pleasant or unpleasant when it was rated with either a score of 1 or 2, or a score of −1 or −2, respectively. The neutral valence corresponded to a null score.

Following the completion of the questionnaire, prior to starting the subsequent stimulation block, the subject was instructed to cleanse their olfactory receptors by smelling a jar filled in with roasted coffee beans; the use of coffee for such a purpose was motivated by empirical evidence on its ability to act as a washout odor.^41^ The whole test session lasted approximately 25 min.

## III. RESULTS

### A. Subjective hedonic classification of the odors

The subjective classification of the odors' hedonic valence was consistent across the majority of the subjects. Indeed, the thermal water was regarded as unpleasant by 73% of the participants (while 18% and 9% of them considered it pleasant or neutral, respectively); the orange essential oil was evaluated as pleasant by 86% of the participants (while 5% and 9% of them marked it as unpleasant or neutral, respectively); the mineral water was regarded as neutral by 77% of the participants (while 18% and 5% of them considered it pleasant or unpleasant, respectively).

**FIG. 2.**
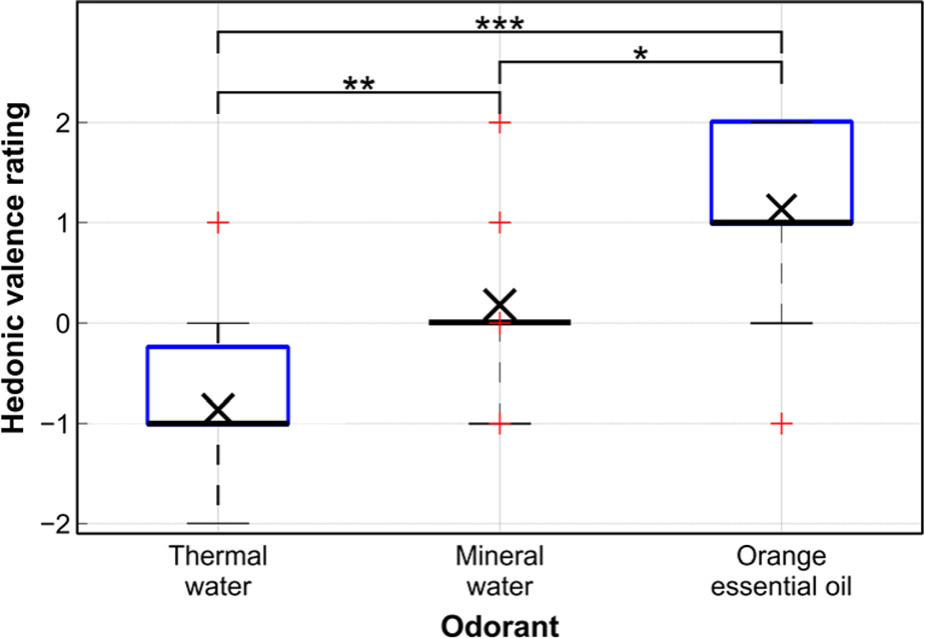
Boxplots of the hedonic valence rating assigned by the subjects to each odorant. Statistically significant differences are marked with asterisks (see the text for statistical outcomes). The symbol × represents the mean value.

In order to assess whether the hedonic valence ratings given by the subjects to the odors had statistically significant differences, an ANOVA test (MATLAB function “anova”) and a pairwise comparison (MATLAB function “multcompare,” using the Bonferroni correction) were performed. Figure 2 presents the outcomes.

All comparisons among the ratings showed statistically significant differences. In particular, the ratings of the orange essential oil and thermal water had a mean difference of 2, with p < 0.001 and a very large effect size (Cohen's d = 2.21); the ratings of the orange essential oil and mineral water had a mean difference of 0.96, with p < 0.05 and a large effect size (Cohen's d = 1.24); the ratings of the thermal water and mineral water had a mean difference of 1.05, with p < 0.01 and a large effect size (Cohen's d = 1.36).

### B. Grand average hemodynamic responses to the olfactory stimuli

**FIG. 3.**
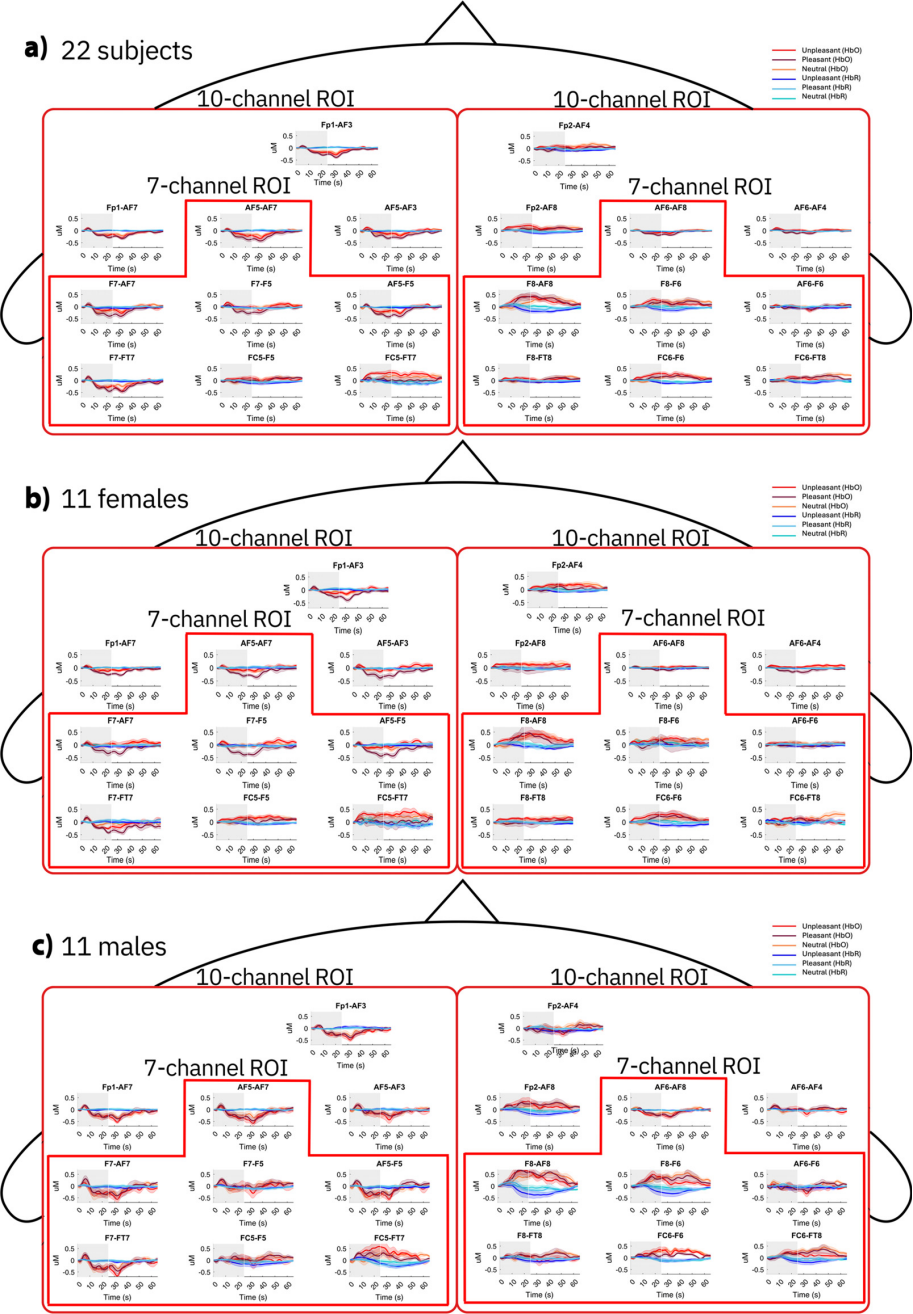
Grand average HbO and HbR signals in response to independent 25 s-long olfactory stimulations with the three odors (unpleasant, neutral, and pleasant). The grand averages were separately determined for each channel and for the following groups of subjects: (a) all 22 participants; (b) only the subset of 11 females; and (c) only the subset of 11 males. The shaded gray area overlying each signal identifies the interval between the onset and offset of the olfactory stimulation. The channels' layout corresponds to the montage shown in Fig. 1(c). The ten- and seven-channel ROIs are identified by surrounding their channels with distinct continuous lines, in each hemisphere.

Grand average HbO and HbR signals are presented in Fig. 3, where they are grouped according to the following three categories of subjects: all 22 participants, only the subset of 11 females, and only the subset of 11 males. In each case, the signals are grouped according to both the ten- and the seven-channel ROIs.

Section III C presents a statistical analysis of the fNIRS data, for the two ROIs.

### C. ROI-based statistical analysis

#### 1. ANOVA on the ten- and seven-channel ROIs

For all 22 participants and each ROI, an ANOVA on the HbO and HbR responses was carried out (using the software JASP, version 0.18.3.0), by considering the following variables: (1) condition of the olfactory stimulation (unpleasant, neutral, and pleasant), briefly referred to in the following as “condition”; (2) hemisphere where the response was detected (left and right), indicated in the following as “hemisphere”; and (3) sex of the subject, indicated in the following as “sex.” The significance level was set at 0.05.

Below, results are presented only for the HbO responses, as the ANOVA applied to HbR responses did not show any statistically significant effect. This outcome was consistent with previous studies that had reported more significant effects for HbO compared to HbR.^42–45^ Furthermore, it is worth noting that the use of HbO over HbR was surveyed in a recent review by Kinder *et al.*,^46^ where justifications for preferring HbO were categorized into five groups: greater sensitivity to cerebral blood flow changes, greater sensitivity to task-evoked changes, higher signal-to-noise ratio, stronger correlation with the fMRI BOLD response, as well as common practice.

For the ten-channel ROI, the ANOVA showed a significant main effect of condition [F(1,43) = 15.785; p < 0.001; η^2^ = 0.027] and sex [F(1,43) = 4.600; p < 0.05; η^2 ^= 0.004], while there was no effect of hemisphere. The interaction of sex with condition also revealed a significant effect [F(1,43) = 9.989; p < 0.01; η^2 ^= 0.017].

For the seven-channel ROI, the analysis showed a significant main effect of condition [F(1,43) = 12.814; p < 0.001; η^2^ = 0.032] and sex [F(1,43) = 5.531; p < 0.05; η^2^ = 0.007], while there was no effect of hemisphere. The interaction of sex with condition also highlighted a significant effect [F(1,43) = 8.082; p < 0.001; η^2^ = 0.020].

**FIG. 4.**
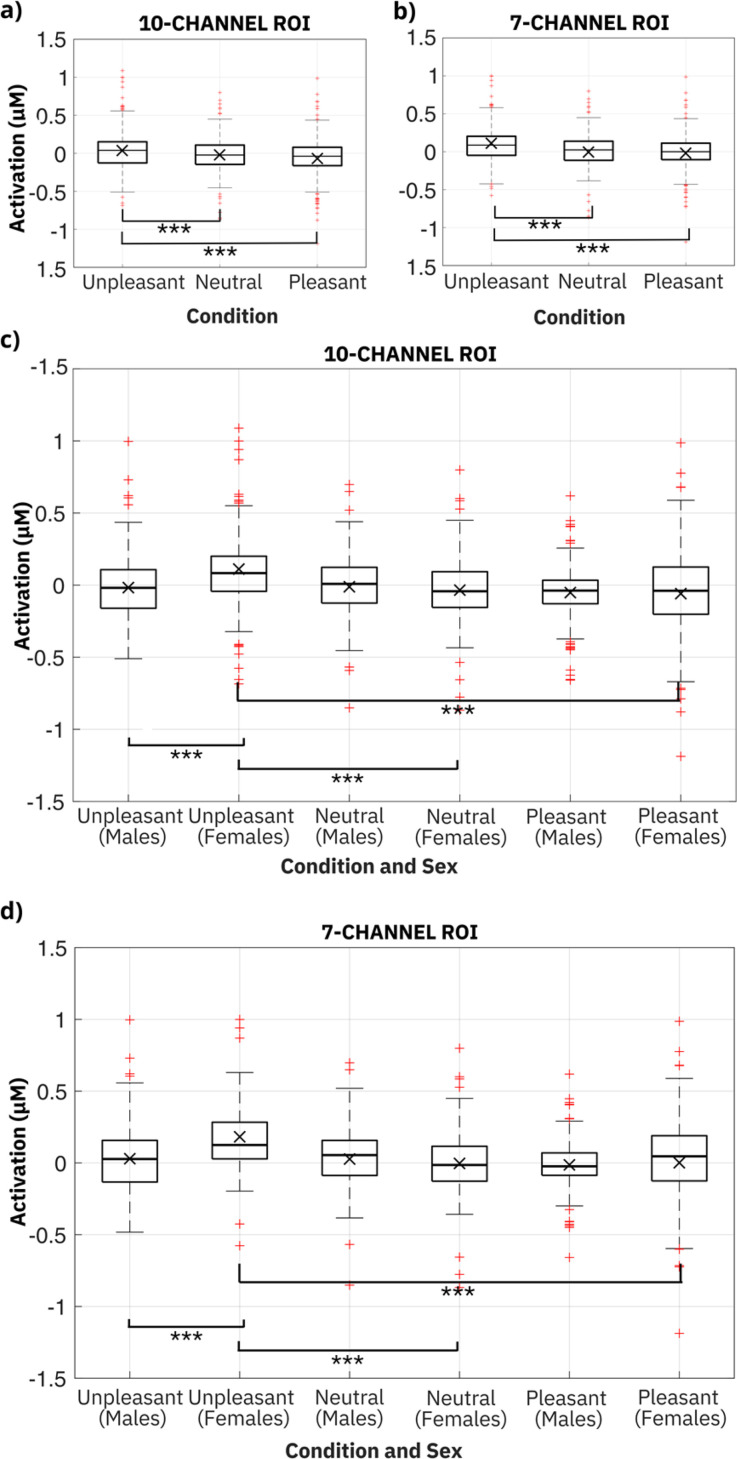
Boxplots of the activation for the three conditions [panels (a) and (b)] and all possible interactions of condition and sex [panels (c) and (d)]. The data were gathered from the ten-channel ROI [panels (a) and (c)] and the seven-channel ROI [panels (b) and (d)]. Statistically significant differences in activation are marked with asterisks (see the text for statistical outcomes). The symbol × represents the mean value.

Following the ANOVA, post-hoc test comparisons, adjusted using the Tukey method, were conducted. The results are presented in Fig. 4, which shows (separately for the ten- and the seven-channel ROIs) boxplots of the activation, defined as the time-averaged amplitude of the grand average HbO signal with the time interval 2–25 s (Fig. 3). In particular, the activation was determined in two cases: first, considering all 22 participants (regardless of their sex) and varying the condition [Figs. 4(a) and 4(b)]; second, varying both sex and condition, so as to study all their possible interactions [Figs. 4(c) and 4(d)]. The outcomes are reported below.

#### 2. Post-hoc analysis on the ten-channel ROI

Figure 4(a) presents the results of post-hoc test comparisons (adjusted using the Tukey method, with a significance level set at 0.05) on condition, from the ten-channel ROI. The data show that, for all 22 participants (regardless of their sex), larger activations were recorded for the unpleasant odor, relative to both the pleasant odor (mean difference = 1.025 × 10^−1^ *μ*M; t-value = 5.496; p_tukey < 0.001), with a small effect size (Cohen's d = 0.370), and the neutral odor (mean difference = 0.701 × 10^−1^ *μ*M; t-value = 3.761; p_tukey < 0.001), with a small effect size (Cohen's d = 0.279).

In order to highlight how the participants' sex affected such outcomes, Fig. 4(c) presents the results of post-hoc test comparisons (adjusted using the Tukey method) on the interaction between condition and sex. The data reveal that the female subjects had greater activations relative to males for the unpleasant odor (mean difference = 1.285 × 10^−1^ *μ*M; t-value = 4.871; p_tukey < 0.001).

Moreover, while in males no statistically significant difference in activation among the odors was detected, in females the unpleasant odor caused larger activations, as compared to both the neutral condition (mean difference = 1.459 × 10^−1^ *μ*M; t-value = 5.447; p_tukey < 0.001) and the pleasant one (mean difference = 1.704 × 10^−1^ *μ*M; t-value = 6.360; p_tukey < 0.001).

However, even in the female subjects, no difference in activation was detected between the pleasant and neutral odors. This was likely due to a similarity in the positive significance attributed in both cases to the olfactory experience, as reported in Sec. IV.

#### 3. Post-hoc analysis on the seven-channel ROI

Figure 4(b) presents the results of post-hoc test comparisons (adjusted using the Tukey method, with a significance level set at 0.05) on condition, from the seven-channel ROI. As with the ten-channel ROI, the data show that, for all 22 participants (regardless of their sex), larger activations were obtained for the unpleasant odor, relative to both the pleasant odor (mean difference = 1.118 × 10^−1^ *μ*M; t-value = 4.713; p_tukey < 0.001), with a small effect size (Cohen's d = 0.388), and the neutral odor (mean difference = 0.917 × 10^−1^ *μ*M; t-value = 3.956; p_tukey < 0.001), with a small effect size (Cohen's d = 0.361).

In order to highlight how the participants' sex affected such outcomes, Fig. 4(d) presents the results of post-hoc test comparisons (adjusted using the Tukey method) on the interaction between condition and sex. As with the ten-channel ROI, the data reveal that the female subjects showed greater activations relative to males for the unpleasant odor (mean difference = 1.525 × 10^−1^ *μ*M; t-value = 4.546; p_tukey < 0.001).

Furthermore, as with the ten-channel ROI, males did not exhibit any statistically significant difference in activation among the odors, while females showed for the unpleasant odor larger activations in comparison to both the neutral one (mean difference = 1.854 × 10^−1^ *μ*M; t-value = 5.410; p_tukey < 0.001) and the pleasant one (mean difference = 1.807 × 10^−1^ *μ*M; t-value = 5.272; p_tukey < 0.001).

As with the ten-channel ROI, even in the female subjects, no difference in activation was detected between the pleasant and neutral odors. An interpretation is provided in Sec. IV.

## IV. DISCUSSION

### A. Higher activations for the unpleasant odor compared to the pleasant one

The first result of this study is the evidence of a statistically significant higher increase in HbO concentrations for the unpleasant odor, as compared to the pleasant one. This was gathered (from each ROI) across the entire pool of 22 subjects [Figs. 4(a) and 4(b)], as well as within the female subgroup [Figs. 4(c) and 4(d)].

This outcome is consistent with the literature, which shows that unpleasant odors typically induce stronger effects; these have been detected not only at a cortical level with fNIRS^16–20^ and EEG^10–13,32,47,48^ but also with deeper fMRI investigations,^4,6,26,35^ as well as through different physiological measurements, such as skin conductance and electrocardiography.^49,50^

The predominant effect of unpleasant odors may be interpreted according to stronger emotional responses that they can elicit, as the human brain tends to create deeper and more enduring memories of unpleasant odors.^51^ Indeed, unpleasant odors might activate more robust neural networks involved in emotional processing, leading to more substantial activation of the OFC and adjacent areas.^22^

### B. Lack of difference in activation between the neutral and pleasant odors

In our experiments, odors of neutral or pleasant valence generated comparable activations, without any statistically significant difference. Their activations differed only from those induced by the unpleasant odor, which were higher (both in the whole group of 22 subjects and in the female subgroup).

We speculate that the similarity in activation for the odors considered as neutral and pleasant may reflect a shared positive, possibly rewarding, significance attributed in both cases to the olfactory experience, irrespective of the odor classification. For instance, it could derive in both cases from an equal sensation of freshness caused by the nebulization of a water-based solution. Indeed, as reported above, while mineral water was regarded as neutral by 77% of the participants, 18% of them considered it pleasant.

### C. Lack of difference in activation between the two hemispheres

This study did not find any statistically significant difference between the two hemispheres in their activations, for each type of odor. In other words, the response of the OFC and adjacent areas to olfactory stimuli with opposite hedonic valence did not show any lateralization effect.

Therefore, this finding is not consistent with studies that have suggested a hemispheric preference in olfactory processing, according to data gathered by fNIRS^16,18,19^ or fMRI.^4,21,22^

Vice versa, our finding agrees with indications that olfactory processing can occur in both hemispheres without any significant preference, as assessed by some fNIRS^17^ and fMRI^26^ studies. Such a view that both the right and left hemispheres are equally involved in olfactory processing is supported by the bilateral representation of olfactory information in the brain.

Nevertheless, it is worth noting that the lack of lateralization observed in this and other fNIRS investigations may not necessarily be due to the absence of an effect, but, rather, to an insufficient ability to detect small differences, as compared to the superior performance of fMRI. Therefore, in our opinion, this result cannot be taken as conclusive.

### D. Differences between the two sexes

This study showed clearly distinct responses according to the participants' sex.

These findings are consistent with previous questionnaire-based studies reporting that females tend to have a heightened sensitivity to the hedonic valence of odors,^27–30^ and previous EEG studies describing that females can exhibit larger signal amplitudes.^31^

Such a higher olfactory sensitivity shown by females could be due to several physiological differences between the two sexes. For instance, in females, the olfactory bulb has been found to be richer in neurons and glial cells, relative to males.^52^ Furthermore, cortical connections involving the OFC with primary olfactory areas have been reported to be stronger in females, compared to males, and to be different based on odors' valence.^53^

To the best of our knowledge, our results provide the first evidence that, like EEG, even fNIRS is able to capture differences between the two sexes in the hedonic evaluation of odors. Therefore, both EEG and fNIRS can be used as low-cost and easy-to-use technologies to obtain quantitative evaluations of, respectively, early and late response components to olfactory stimulations, whose different effects in males and females have typically been investigated qualitatively through questionnaires.^27–30^

### E. Possible interplay between perceived hedonic valence and perceived arousal

In this study, the participants were asked to rate each odor exclusively in terms of perceived hedonic valence, which refers to its positive or negative emotional value. However, an odor might also be rated in terms of perceived arousal, which refers to the elicited level of activation or alertness, from low (calming) to high (stimulating). As both dimensions contribute to the overall perceptual experience, future investigations might elucidate whether possible differences in arousal can significantly affect fNIRS detectable cortical activations in response to odors with different hedonic valence.

## V. CONCLUSIONS AND FUTURE DEVELOPMENTS

Using fNIRS, we measured cortical activity over the OFC and adjacent areas in response to olfactory stimulation with two odors having opposite hedonic valence. The results showed significantly higher increases in HbO concentration during exposure to the unpleasant odor in comparison to the pleasant one. No differences in activation were found between the two hemispheres. Conversely, different responses were obtained from the two sexes, since only the female subgroup exhibited significantly higher activations for the unpleasant odor.

These outcomes indicate that fNIRS is adequately able to capture peculiarities of cortical activations elicited by olfactory stimuli, in terms of differences between pleasant and unpleasant odors, and differences between males and females.

This evidence sets fNIRS next to EEG in terms of technologies that can gather meaningful information on cortical activations due to olfactory stimuli, with lower costs and easier operation (but also lower resolution), compared to fMRI. Nevertheless, information gathered by fNIRS and EEG is complementary, as each of them is better suited to detect early and late response components, respectively.

Future developments of this work might be focused on testing more odors, spanning a broader range of hedonic valence, and investigating possible effects of odor familiarity, intensity, and arousal.

Furthermore, it would be interesting to use fNIRS to investigate olfactory responses in subjects with diseases that can directly or indirectly alter olfactory responses. For instance, preliminary fNIRS studies have shown that individuals with Autism Spectrum Disorder show alterations in olfactory processes,^54^ and patients with Multiple Chemical Sensitivity have blood flow alterations in the prefrontal cortex during exposure to olfactory stimuli.^55,56^ Moreover, other studies are investigating the potential efficacy of detecting by fNIRS alterations in the oxygenation of the OFC during olfactory stimulation for an early diagnosis of Mild Cognitive Impairment and Alzheimer's Disease.^57^

## VI. METHODS

### A. Olfactory stimulation system

The olfactory stimulation system consisted of three independent mesh nebulizers and a microcontroller. Each nebulizer (Gima S.p.A., Italy) was activated by a relay (KF-301, AZ Delivery, Germany), whose control signal was provided by a microcontroller (Arduino Micro, Arduino, Italy). A custom script on the microcontroller, combined with a MATLAB custom script, allowed for activating the three nebulizers according to any desired sequence. In order to synchronize the nebulizers' activation with the fNIRS signal acquisition, the MATLAB script sent trigger signals to the acquisition software (NIRStar, NIRx Medical technology, Germany) via the software Lab Streaming Layer, which was used for data streaming.^58^

### B. Subjects

Thirty-two adult subjects (18 females and 14 males), aged between 19 and 34 years (mean age 26.5 years), were initially recruited. The only recruitment criteria were the absence of diagnosed neurological diseases and the absence of known olfactory sensation impairments. Nine subjects were smokers. All participants were informed about the purpose and protocol of the study. No personal information was collected.

After the tests, the data related to ten subjects were discarded, due to insufficient quality, as described in the following of the paper. Therefore, the subsequent analysis was performed on 22 subjects, of which 11 were females and 11 were males.

### C. Preparation of the subjects, administration of the odors, and acquisition of the fNIRS signals

The subject's head was fitted with an fNIRS cap (NIRScap) available from the used fNIRS system. The cap was equipped with 16 optodes (eight for each hemisphere), according to the montage shown in Fig. 1(c). The optodes' localization on the scalp adhered to the 10–5 system, with inter-optode distances of approximately 3 cm.

Before testing, the subjects were instructed to blow their nose using provided tissues, sit comfortably on a chair, and maintain a relaxed state, keeping their eyes closed throughout the test (to minimize motion artifacts), as represented in Fig. 1(b).

The mesh nebulizers were held at 10 cm from the subject's nostrils. The subjects were asked to focus on the presented odor while breathing naturally, without sniffing the odor.

The fNIRS signals were measured using a commercial instrument (NIRscout, NIRx Medical technology, Germany) and its proprietary software (NIRStar), which used 760 and 850 nm wavelengths. Data were collected at the system's preset sampling frequency of 7.81 Hz.

To verify, before testing, that all channels were able to provide adequate signal quality (i.e., a sufficiently high signal-to-noise ratio in the optical coupling between source and detector), a signal optimization routine (calibration) was conducted for all channels. The calibration was performed via NIRStar, which acquired a short data segment and evaluated a signal quality metric, according to a noise level, defined by a coefficient of variation (CV): any channel was marked as “excellent” if CV < 2.5%, “acceptable” if 2.5≤CV ≤ 7.5%, or “critical” if CV > 7.5%. A signal quality map allowed the operator to visually inspect the quality of all channels, according to a color coding: green for “excellent,” yellow for “acceptable,” red for “critical,” as well as white if the signal was lost and the noise could not be calculated. The quality of each channel was considered adequate if it had been marked as “excellent” or “acceptable.” For any channel with poor quality, the arrangement of the corresponding optodes was adjusted, until adequate quality was achieved.

To ensure synchronization between the fNIRS recordings and the electrical signals controlling the nebulizers, the “Data streaming” feature of the NIRStar software received trigger signals from a MATLAB script that managed the nebulizers. This allowed for recording the instant corresponding to the olfactory stimulus' onset (release of the odorant from the nebulizer).

### D. Exclusion of low-quality channels

Following the recording of the fNIRS responses to the olfactory stimulations, data quality assessment and subsequent pre-processing were conducted using the software MATLAB, with custom scripts and functions from the Homer2 package.^59^

In particular, data quality was assessed using the Homer2 function “enPruneChannels,” which removed signals with excessively high or low values. The used threshold values were those recommended by the manufacturer (NIRx Medical technology): a signal-to-noise ratio threshold of 6 and a signal range of 0.09–1.4 V. Visual inspection was also conducted to evaluate the signal quality.

Any participant was excluded from further analysis if they showed poor-quality signals in more than 8 out of the total 20 channels for the ten-channel ROI, and in more than 5 out of the total 14 channels for the seven-channel ROI. As a result, 22 out of 32 subjects initially tested were retained for further analysis.

### E. fNIRS signal pre-processing

According to fNIRS best practices, the signal pre-processing pipeline consisted of the following stages (see Zhou *et al.*^60^ for a useful graphical summary).

As a first step, the intensity data were converted into optical densities, using the MATLAB function “hmrIntensity2OD.”

As a second step, motion artifacts were corrected using the Temporal Derivative Distribution Repair algorithm.^61^ Subsequently, the MATLAB function “hmrMotionArtifact” was used to identify and remove trials affected by residual motion artifacts, which were identified as changes in signal amplitude of 0.4 V or more within a temporal window of 1 s, or as 13-folds or larger changes in standard deviation, within 1 s.

As a third step, a bandpass filter (Butterworth, third order, 0.005–0.2 Hz) was applied to the optical densities via the MATLAB function “hmrBandpassFilt” to reduce both slow drifts and high-frequency noise, while preserving activity within the frequency band of interest.

As a fourth step, the optical densities were converted into hemoglobin concentration changes, by using the MATLAB function “hmrOD2Conc,” according to the modified Beer–Lambert law; to that aim, we employed a differential path length factor of 6 m and the following absorption coefficients: *μ*a(HbO, 760 nm) = 1486.6 M^−1^ cm^−1^, *μ*a(HbO, 850 nm) = 2526.4 M^−1^ cm^−1^, *μ*a(HbR, 760 nm) = 3843.7 M^−1^ cm^−1^, and *μ*a(HbR, 850 nm) = 1798.6 M^−1^ cm^−1^.

As a fifth step, for each subject, bad channels (identified as described in Sec. VI D) were removed from the analysis.

For every condition and every channel, the signals that had been gathered from any given subject by repeating the olfactory stimulations across the four trials [Fig. 1(e)] were block-averaged, using the MATLAB function “hmrBlockAvg.” Baseline correction was performed using a window of 2 s before the onset of each stimulus. The resulting signals from all subjects were then averaged (using a custom MATLAB script), to obtain a grand average signal.

## Data Availability

The data that support the findings of this study are available from the corresponding author upon reasonable request.
